# Respiratory syncytial virus in adults aged ≥ 50 years in France: a database and time-series modelling analysis (2014–2020)

**DOI:** 10.1007/s15010-026-02799-7

**Published:** 2026-06-01

**Authors:** Rachel M. Reeves, Saskia Hagenaars, Rita Ammoun, Bénédicte Borsik, Xavier Colin, Maria João Fonseca, Triantafyllos Pliakas, Emanuele Amodio, Colin A. Russell, Richard Osei-Yeboah, Alen Marijam, Paul Loubet

**Affiliations:** 1 GSK, 2929 Walnut Street, Ste. 1700, Philadelphia, PA 19104 USA; 2 IQVIA Solutions, Amsterdam, The Netherlands; 3 IQVIA, Courbevoie, France; 4 IQVIA, Bordeaux, France; 5 GSK, Rueil-Malmaison, France; 6 GSK, Lisbon, Portugal; 7 GSK, Wavre, Belgium; 8 Impact Epilysis, Thessaloniki, Greece; 9 Department of Health Promotion, Mother and Child Care, Internal Medicine and Medical Specialties, University of Palermo, Palermo, Italy; 10 Department of Medical Microbiology and Infection Prevention, Amsterdam University Medical Centre, Amsterdam, The Netherlands; 11 Centre for Global Health, Usher Institute, The University of Edinburgh, Edinburgh, UK; 12 Virulence Bactérienne et Infections Chroniques, INSERM U1047, Univ Montpellier, Service des Maladies Infectieuses et Tropicales, CHU Nîmes, Nîmes, France

**Keywords:** RSV, Influenza, Time-series modelling, Older adults, Hospitalisation

## Abstract

**Purpose:**

This study aimed to quantify the burden of respiratory syncytial virus (RSV)- and influenza-attributed acute respiratory infections (ARI) in older adults in France. Modelling was applied to real-world data on ARI-attributed hospital admissions and in-hospital mortality to estimate pathogen-specific outcomes and investigate the underreporting of RSV in standard clinical practice. Additionally, healthcare resource utilisation was assessed.

**Methods:**

This retrospective, observational cohort study used routinely collected, secondary care data from the Programme National de Médicalisation des Systèmes d’Information (PMSI) database. All patients aged ≥ 50 years with an ARI-related hospitalisation, identified through International Classification of Diseases, 10th Revision (ICD-10) coding, between July 2014 and February 2020 were included. The predicted number and annual rate of hospitalisations were calculated using a Quasi-Poisson regression model, stratified by age group. Sensitivity analyses were used to investigate cardiorespiratory hospital admissions and in-hospital mortality.

**Results:**

2,009,218 patients with an ARI-related hospitalisation were identified. Predicted rates of RSV- and influenza-attributable ARI hospitalisations ranged from 0.05 and 0.27 per 1000 person-years among 50–54-year-olds to 14.38 and 12.48 per 1000 person-years among ≥ 90-year-olds, respectively. Rates of in-hospital mortality were similar between the two pathogens, while RSV was predicted to account for a much larger number of cardiorespiratory hospitalisations than influenza. RSV-coded hospitalisations lasted 8–11 days across age groups, and 25.6% of 55–59-year-olds hospitalised with RSV were admitted to an intensive care unit.

**Conclusion:**

These data reveal a substantial burden of RSV-related morbidity and mortality among older adults in France, demonstrating a need to prioritise RSV prevention.

**Supplementary Information:**

The online version contains supplementary material available at 10.1007/s15010-026-02799-7.

## Introduction

Respiratory syncytial virus (RSV) represents a significant burden in France and is a leading cause of acute respiratory infection (ARI), especially among newborns, the older population and those with comorbidities [1–3]. Within these groups, RSV has a significant effect on morbidity and mortality [1, 4, 5].

In France, as well as in other European countries, the true burden of RSV in older adults is not fully understood [6–9]. This is partly due to a lack of routine RSV testing, which has led to limited RSV-specific diagnoses coded in routinely collected databases. Recently, a nationwide analysis of RSV-related hospitalisations in adults in France between 2012 and 2021 showed a significant increase in the number of RSV-related hospitalisations over time, likely due to an increase in routine RSV testing and improved coding practices [6]. This study demonstrated that RSV represents a significant cause of ARI, specifically lower respiratory tract disease (LRTD). RSV-related LRTD was found to lead to prolonged hospitalisation, considerable rates of intensive care unit (ICU) admissions, frequent readmission to hospital and a rate of mortality comparable to that of influenza [6]. Nonetheless, estimates of RSV infection and associated morbidity and mortality based on International Classification of Diseases, 10th Revision (ICD-10) RSV diagnosis codes alone have been shown to significantly underestimate the true burden of RSV disease in other countries [10, 11] .

To overcome the limitation of a lack of routine RSV testing, modelling strategies have been used pragmatically to estimate the true burden of RSV [2, 12–14]. Recently published estimates of RSV hospitalisation in older adults in Europe, adjusted for diagnostic testing, clinical specimens and case definitions, estimated annual RSV hospitalisation rates in adults aged ≥ 60 years in France to be 289 per 100,000 person-years [15]. However, a detailed breakdown by age was not reported [15]. Multiple regression time-series modelling (TSM) is one strategy that has been frequently used to generate detailed estimates of the burden of influenza, RSV and other respiratory viruses. TSM uses the seasonal variation in respiratory virus circulation to attribute outcomes (e.g. hospitalisation, mortality or primary care consultation) to specific viruses [14]. While this methodology has been employed across multiple European countries for RSV [13, 16, 17], there are limited studies in older adults in France [2]. In light of the recent availability of RSV vaccines for older adults [18–20], accurate estimation of the true burden of disease associated with RSV is essential to inform decision-making on preventative measures, including use in cost-effectiveness modelling of RSV vaccines.

This study aimed to fill critical gaps in knowledge by providing a detailed and recent analysis of the secondary care burden of RSV in adults aged ≥ 50 years in France, with analysis of patient and clinical characteristics and healthcare resource use (HCRU) based on ICD-10-coded admissions, contextualised against all ARI admissions. In addition, this study applied TSM methodology to generate detailed age-specific estimates of the burden of RSV in older adults in terms of LRTD- and cardiorespiratory-related hospital admissions and in-hospital mortality in France.

## Methods

### Study design and data sources

This non-interventional retrospective cohort study was based on the secondary use of routinely collected data in France, using the Programme National de Médicalisation des Systèmes d’Information (PMSI) database. The PMSI database covers all hospital stays in France, with diagnoses coded as primary (PD), related (RD) and associated (AD). In the PMSI database, PD refers to the healthcare issue that led to hospital admission, while RD refers to a condition that has a direct link to the PD, such as a complication or contributing factor, and is only recorded when the ICD-10 code of the PD is in the Z chapter (e.g. palliative care, surveillance or chemotherapy session). AD refers to coexisting conditions that are not directly related to the reason for admission but influence the patient’s overall care (e.g. diabetes, hypertension). Data on the date of hospital admission, duration of stay and in-hospital mortality are also recorded.

ARI-coded hospitalisations (ARI as either a primary or secondary diagnosis code [see Supplementary File 1 for a list of relevant ICD-10 codes]) between 1st July 2014 and 29th February 2020 were included in the study, equating to six RSV seasons in France. The index date was the date of the first day of the first ARI-coded hospitalisation. Outcomes were assessed up to 90 days after each hospitalisation. A sensitivity analysis was performed using an expanded cardiorespiratory definition, as RSV infections have been linked to various cardiovascular outcomes [21]. For sensitivity analyses of cardiorespiratory-coded hospital admissions, the index date was the date of the first day of the first cardiorespiratory hospital admission. Patients were followed from the respective index date until the earliest of: date of in-hospital death, end of study period, or date of latest available data.

### Study population and outcomes

The study population included all patients in the PMSI database with an ARI-coded hospital admission and aged ≥ 50 years at the index date. All ARI-coded hospitalisations, including pathogen-specified (RSV, influenza and other pathogens) and unspecified, with ARI as either PD, RD or AD, were assessed. Cardiorespiratory-associated hospital admissions were also assessed for the sensitivity analyses, with cardiorespiratory diagnosis codes as either PD, RD or AD. Patients were excluded if they experienced a hospitalisation that could be captured as a “dialysis session”, which represents recurrent stays not typically related to a hospitalisation of interest.

Four coded diagnosis groups were defined and analysed. These included patients with any ARI (pathogen-specific and non-pathogen specific), patients with any pathogen-specific ARI (including but not limited to RSV and influenza), patients with RSV-specific ARI, and patients with influenza-specific ARI (Supplementary File 1). These groups were not mutually exclusive, and all diagnosis codes within a patient’s record were used, so patients could be categorised into more than one of these ARI diagnosis subgroups. These diagnostic subgroups were further stratified by age and type of ARI (upper respiratory tract disease [URTD] and LRTD).

Comorbidities recorded within the PMSI health record (i.e. during hospitalisation) were identified for all patients. Patients were then considered high-risk if they displayed one or more of the following comorbidities of interest: chronic obstructive pulmonary disease, asthma, any other chronic respiratory or pulmonary disease, chronic heart failure, advanced liver or renal disease and/or diabetes. Other comorbidities described included stroke and immunocompromised status, including immunocompromised subtype. These comorbidities were selected based on relevant literature regarding the risk factors for RSV in older adults [1, 5, 22–24]. A patient was considered to have a comorbidity at the time of an admission if there was at least one ICD-10 code for that condition within their entire medical history within the PMSI database, up to and including the date of that hospital admission.

### Statistical methods

Patient demographics, clinical characteristics and comorbidities were described at index date, with variables of interest presented as summary statistics at the patient-level following first hospital admission.

For each of the four diagnostic groups, the observed total number and annual rate of RSV- and influenza-coded ARI or cardiorespiratory hospital admissions, as well as admission-level in-hospital mortality (all-cause and/or pathogen-specific) were calculated. In-hospital mortality refers to those patients who died during an ARI-related hospital admission, as recorded on the death certificate, where cause of death is ARI-related.

#### Time-series modelling

Due to the high proportion of ARI admissions categorised as LRTD, TSM was conducted for the LRTD subgroup only, and for the cardiorespiratory sensitivity analysis.

The number and annual rate of RSV- and influenza-linked LRTD hospital admissions among adults aged ≥ 50 years were estimated using TSM, overall and by age group. Quasi-Poisson regression models with an identity link function and 12 monthly timepoints per year were used for the total population of adults aged ≥ 50 years. The predictors in the time-series model were levels of RSV and influenza laboratory confirmations taken from the Géodes data from the French Public Health Agency, as proxies for the circulation of the two viruses [25]. For RSV, the external predictor variable was the monthly rate of SOS Médecins consultations for bronchiolitis in children under 2 years old (for 10,000 consultations). For influenza, the external predictor variable was the monthly rate of consultations for influenza in adults aged ≥ 65 years.

The base model was fitted using the surveillance data from Géodes, with a stepwise model-building approach applied to incorporate seasonal components to account for periodic variation. To account for delays in pathogen circulation between age groups (i.e. RSV laboratory confirmations in children under 2 years old and hospital admissions in the aged ≥ 50 years population), and given the monthly granularity of the data, a one-month lag was tested for RSV and for influenza. Time trends, including linear, seasonal and secular, were explored to account for temporal variation over the study period. The final model was selected based on the lowest quasi-Akaike Information Criterion (qAIC) value.

Estimates of the number of hospital admissions attributable to each pathogen of interest were calculated by multiplying the coefficient from the time-series model by the total number of laboratory confirmations. For annual rates of hospital admissions, the numerator for each year was obtained by summing the estimated monthly number of admissions for a given pathogen of interest. The denominator populations were based on French national population estimates. The French population of adults aged ≥ 50 years and who fulfilled all inclusion criteria was derived from the Institut National de la Statistique et des Études Économiques (INSEE) population of each year in France.

The upper and lower exact Poisson confidence intervals (CI) for the annual rates were estimated using the following formula, where CINV() is the *p*th quantile from the chi-squared distribution with degrees of freedom d, CINV(p, d):

1 $$\text{Exact Poisson CI}=(\mathrm{CINV}(0.05/2, 2*\text{total number of events in the study population})/2)/\text{total person-years in time period in the database denominator population},\mathrm{CINV}(1-0.05/2, 2*\text{total number of events in the study population}+1)/2)/\text{total person-years in time period in the database denominator population})$$

The same modelling approach was applied with in-hospital mortality as the outcome, as well as for cardiorespiratory admissions and cardiorespiratory in-hospital mortality.

### Multiplier rates

To represent the extent to which RSV may have been clinically underdiagnosed, annual RSV-attributable LRTD hospitalisation multiplier rates were calculated as the estimated number divided by observed number of RSV-attributable LRTD hospitalisations. The estimated number of hospitalisations was derived from TSM, and the observed numbers were based on ICD-10 coding.

### Healthcare resource utilisation

#### Length of hospital stay

Length of hospital stay in the PMSI database corresponds to the number of nights spent in hospital.

#### Hospital readmission

ARI-related readmission and all-cause readmission were presented as binary variables (yes/no) and patients were considered readmitted if their readmission date to hospital due to ARI/any-cause was within 90 days of the discharge date of the ARI-associated index admission.

### Sensitivity analysis

Cardiorespiratory codes, including all respiratory disease codes and all cardiovascular codes, were used to identify hospital admissions and in-hospital deaths in the sensitivity analysis. This included ICD-10 codes I00-99 and J00-99.

## Results

### Study demographics and clinical characteristics

Among adults aged ≥ 50 years hospitalised with any ARI during the study period, the median age at first admission was 79.0 years (Table 1). Age varied slightly across diagnosis groups, with patients admitted for RSV-specific ARI being the oldest (median age 80.0 years), followed by those with influenza-specific ARI (median age 78.0 years) and pathogen-specific ARI (median age 77.0 years).

**Table 1:** Demographics and clinical characteristics for the any ARI, pathogen-specific ARI, RSV-specific ARI and influenza-specific ARI groups at first admission during the study period

Demographic characteristics		Any ARI	Pathogen-specific ARI	RSV-specific ARI	Influenza-specific ARI
Number of patients	N	2,009,218	646,748	7459	97,109
Age at index date^a^	Mean (SD)	76.9 (12.18)	75.6 (12.33)	77.7 (12.01)	76.0 (12.20)
	Median (Q1–Q3)	79.0 (67.0–87.0)	77.0 (66.0–86.0)	80.0 (69.0–87.0)	78.0 (67.0–86.0)
Age group at index date,^a^ n (%)	Overall ≥ 50	2,009,218 (100.0)	646,748 (100.0)	7459 (100.0)	97,109 (100.0)
	Overall ≥ 60	1,788,679 (89.0)	563,607 (87.1)	6774 (90.8)	85,019 (87.6)
	50–54	92,998 (4.6)	35,764 (5.5)	293 (3.9)	5274 (5.4)
	55–59	127,541 (6.3)	47,377 (7.3)	392 (5.3)	6816 (7.0)
	60–64	165,548 (8.2)	60,139 (9.3)	598 (8.0)	8346 (8.6)
	65–69	202,179 (10.1)	71,532 (11.1)	730 (9.8)	9771 (10.1)
	70–74	205,257 (10.2)	70,446 (10.9)	773 (10.4)	10,330 (10.6)
	75–79	236,807 (11.8)	77,062 (11.9)	837 (11.2)	11,827 (12.2)
	80–84	320,176 (15.9)	97,657 (15.1)	1155 (15.5)	15,812 (16.3)
	85–89	344,986 (17.2)	98,706 (15.3)	1391 (18.6)	15,963 (16.4)
	≥ 90	313,726 (15.6)	88,065 (13.6)	1290 (17.3)	12,970 (13.4)
Gender, n (%)	Female	958,284 (47.7)	296,327 (45.8)	4317 (57.9)	51,377 (52.9)
	Male	1,050,934 (52.3)	350,421 (54.2)	3142 (42.1)	45,732 (47.1)
Follow-up period					
Follow-up from index date to end of follow-up (years)	Mean (SD)	4.6 (2.44)	4.5 (2.50)	3.9 (1.82)	4.5 (1.96)
Type of ARI at index date,^a^ n (%)	URTD	51,509 (2.6)	2297 (0.4)	18 (0.2)	309 (0.3)
	LRTD	1,910,640 (95.1)	597,382 (92.4)	7441 (99.8)	49,731 (51.2)
Presence of comorbidities, n (%)					
High-risk group^b^		1,433,706 (71.4)	455,479 (70.4)	5753 (77.1)	61,136 (63.0)
Chronic respiratory/pulmonary conditions		574,140 (28.6)	124,017 (19.2)	1906 (25.6)	16,878 (17.4)
Cardiac disorders		1,099,358 (54.7)	344,553 (53.3)	4418 (59.2)	46,856 (48.3)
Chronic kidney diseases		316,347 (15.7)	105,582 (16.3)	1541 (20.7)	14,987 (15.4)
Chronic liver diseases		92,900 (4.6)	39,364 (6.1)	351 (4.7)	4388 (4.5)
Diabetes		455,014 (22.6)	149,452 (23.1)	1824 (24.5)	23,645 (24.3)
Stroke		153,697 (7.6)	56,103 (8.7)	559 (7.5)	6517 (6.7)
Immunocompromised		535,349 (26.6)	194,485 (30.1)	2311 (31.0)	22,388 (23.1)
Haematopoietic stem cell transplant		5268 (0.3)	2947 (0.5)	174 (2.3)	443 (0.5)
Solid organ transplantation		24,894 (1.2)	13,184 (2.0)	360 (4.8)	2003 (2.1)
Solid organ malignancies		347,789 (17.3)	122,974 (19.0)	1056 (14.2)	11,081 (11.4)
Haematological malignancies		71,633 (3.6)	30,727 (4.8)	669 (9.0)	3863 (4.0)
Chronic inflammatory diseases (autoimmune diseases)		68,270 (3.4)	23,619 (3.7)	380 (5.1)	3730 (3.8)
End-stage renal diseases		43,916 (2.2)	17,683 (2.7)	280 (3.8)	2679 (2.8)
Other immunodeficiency conditions		134,165 (6.7)	53,308 (8.2)	960 (12.9)	7487 (7.7)
Immunosuppressants		145,881 (7.3)	56,027 (8.7)	717 (9.6)	5009 (5.2)

*ARI* acute respiratory infection, *COPD* chronic obstructive pulmonary disease, *LRTD* lower respiratory tract disease, *Q1* first quartile, *Q3* third quartile, *RSV* respiratory syncytial virus, *SD* standard deviation, *URTD* upper respiratory tract disease

^a^Index date was the first day of the initial ARI-associated hospital admission; ^b^Patients were considered high-risk if they presented co-morbidity with COPD, asthma, other chronic respiratory/pulmonary disease, chronic heart failure, advanced liver or renal disease or diabetes on or before the date of ARI admission

**Fig. 1:**
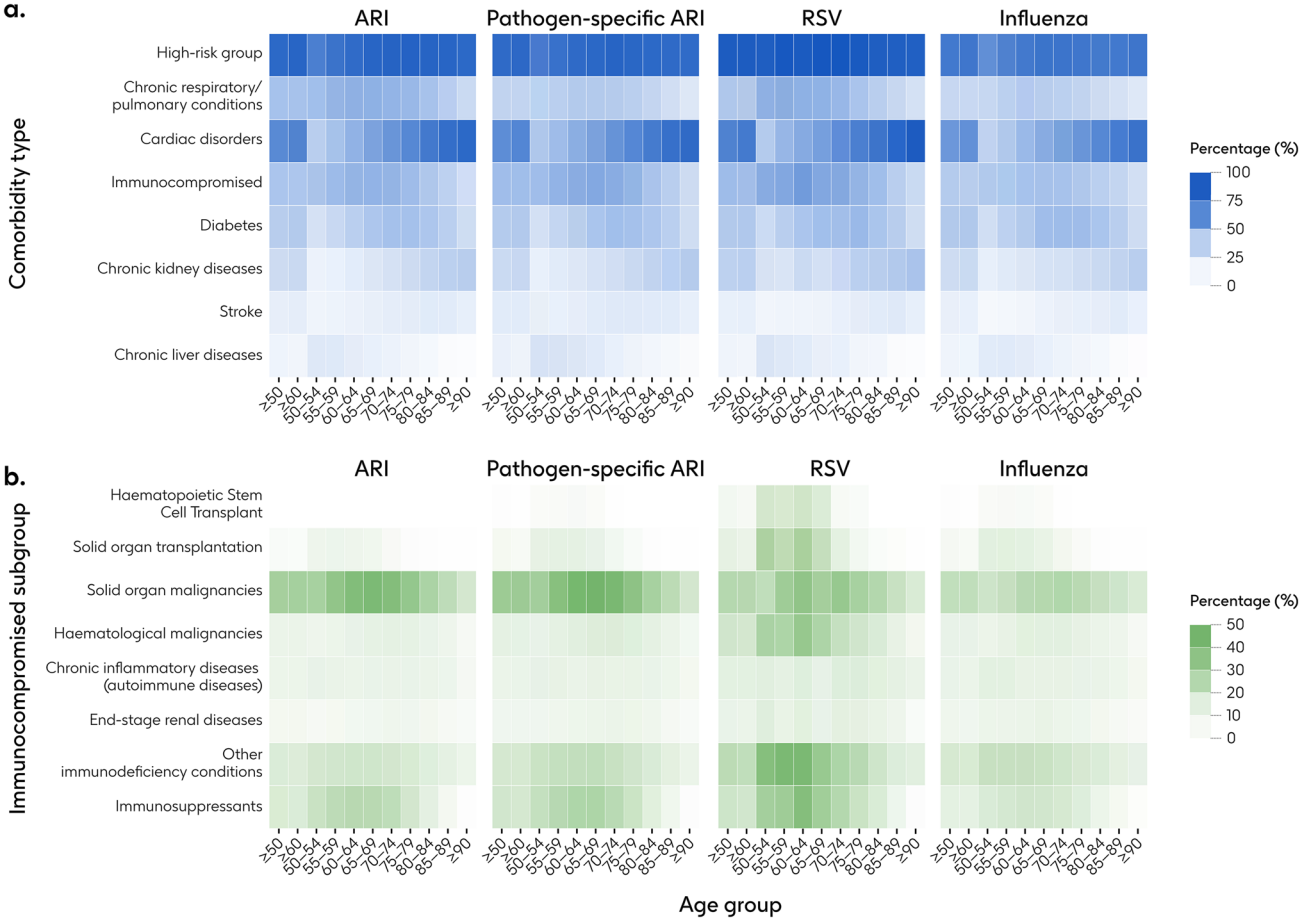
Age-specific comorbidity patterns across diagnosis groups, including general comorbidities (**a**) and immunocompromised subgroup (**b**). Patients were considered high-risk if they presented co-morbidity with COPD, asthma, other chronic respiratory/pulmonary disease, chronic heart failure, advanced liver or renal disease or diabetes on or before the date of ARI admission. *ARI* acute respiratory infection, *COPD* chronic obstructive pulmonary disease, *RSV* respiratory syncytial virus

A high proportion of patients were classified as high-risk at their first hospital admission (Fig. 1), including 71.4% in the overall ARI population, 70.4% among those with pathogen-specific ARI, 63.0% with influenza, and 77.1% with RSV (Table 1). Almost all patients with any ARI, pathogen-specific ARI and RSV-specific ARI were diagnosed with LRTD (95.1%, 92.4% and 99.8%, respectively), with a low proportion of URTD (Table 1). In contrast, only 51.2% of all patients with influenza-specific ARI were diagnosed with LRTD, although 48.5% of these patients did not have a recorded diagnosis of either LRTD or URTD. Of those patients with a recorded diagnosis of LRTD or URTD (n = 50,040), 99.4% were diagnosed with LRTD, similar to the proportion of patients with RSV-specific ARI.

Across all first ARI admissions, including first pathogen-specific ARIs, comorbidities were highly prevalent (Fig. 1). The most common comorbidities among the any ARI subgroup were cardiac disorders (54.7% of patients), chronic respiratory/pulmonary conditions (28.6%) and immunocompromised status (26.6%). Notably, the highest prevalence of cardiac disorders occurred in the RSV-specific ARI group, at 59.2% of admissions (Table 1). These results also demonstrated distinct trends across age groups. Chronic respiratory conditions peaked in younger age groups and showed lower prevalence in the older age groups.

### Observed hospital admissions

The observed rate of ARI hospital admissions per 1000 person-years, based on ICD-10 coding, increased with age. Overall ARI observed hospital admission rates ranged from 27.53 per 1000 person-years in the 50–54 age group to 597.47 per 1000 person-years in those aged ≥ 90 years. For pathogen-specific ARIs, admission rates followed a similar pattern, starting at 10.34 per 1000 person-years in the youngest group and reaching 161.40 per 1000 person-years in individuals aged ≥ 90 years.

Observed RSV-specific ARI admission rates were lower overall but still increased with age, ranging from 0.10 per 1000 person-years in the youngest age group to 2.66 per 1000 person-years in those aged ≥ 90 years.

Observed admission rates for influenza-specific ARIs were higher than RSV across all age groups, ranging from 1.43 per 1000 person-years in the 50–54 age group to 23.14 per 1000 person-years in the oldest population.

### Time-series modelling-estimated hospital admissions

The final model for the overall population aged ≥ 50 years incorporated a 4-week lag for Géodes RSV data, no lag for Géodes influenza data, and seasonal trends. The overall final predictive model was found to fit the observed data well (Supplementary Fig. 1). The model based on all patients aged ≥ 50 years was applied for all other age groups. TSM was performed for LRTD admissions only, given the high proportion (95.1%) of all patients diagnosed with LRTD at the point of hospital admission (Table 1).

Estimated RSV and influenza LRTD hospital admission rates, using TSM, were substantial and similar: 1.86 (95% CI: 0.81–2.92) per 1000 person-years for RSV and 1.89 (95% CI: 1.52–2.27) per 1000 person-years for influenza in the ≥ 50 years population, and 2.71 (95% CI: 1.21–4.22) per 1000 person-years for RSV and 2.68 (95% CI: 2.15–3.21) per 1000 person-years for influenza in the ≥ 60 years population (Table 2). In younger age groups, influenza accounted for the largest proportion of predicted LRTD hospital admissions. For instance, among 50–54-year-olds, 6.16% of LRTD admissions were predicted to be influenza-attributable compared with only 1.21% RSV-attributable. However, this trend was reversed in the oldest age groups (Table 2). Estimated RSV hospital admission rates were particularly high in the 85–89 and ≥ 90 years age groups, at 9.42 (95% CI: 5.19–13.67) and 14.38 (95% CI: 6.46–22.38) per 1000 person-years, respectively. The estimated RSV hospital admission rates in older age groups were substantially higher than those observed, with multiplier rates between 11.14 and 32.28 for adults in the 60–64 years and above age groups (Table 2). The overall pattern of estimated admissions by age matched that of the RSV-coded and overall LRTD admissions in France.

**Table 2:** Total number and annual rate of RSV-associated and influenza-associated LRTD hospital admissions among adults aged ≥ 50 years

Age group	Pathogen	Observed hospital admissions, n	Total predicted hospital admissions, n (Exact Poisson CI)	Predicted admission rates per 1000 person-years (Exact Poisson CI)	Percentage attributable,^a^ %	Multiplier rate: observed to predicted admissions
Overall ≥ 50	RSV	12,201	278,015 (121,273; 435,962)	1.86 (0.81; 2.92)	9.19%	22.79
	Influenza	73,762	283,070 (227,917; 338,867)	1.89 (1.52; 2.27)	9.36%	3.84
Overall ≥ 60	RSV	11,121	268,288 (120,277; 417,477)	2.71 (1.21; 4.22)	9.80%	24.12
	Influenza	66,129	265,332 (213,174; 318,122)	2.68 (2.15; 3.21)	9.70%	4.01
50–54	RSV	423	1351 (NC; 8295)	0.05 (NC; 0.32)	1.21%	3.19
	Influenza	2981	6875 (4499; 9288)	0.27 (0.18; 0.36)	6.16%	2.31
55–59	RSV	657	7889 (NC; 16,563)	0.32 (NC; 0.67)	4.44%	12.01
	Influenza	4652	10,804 (7814; 13,829)	0.44 (0.32; 0.56)	6.07%	2.32
60–64	RSV	997	11,107 (NC; 23,436)	0.47 (NC; 0.99)	4.53%	11.14
	Influenza	6394	15,717 (11,469; 20,016)	0.67 (0.49; 0.85)	6.42%	2.46
65–69	RSV	1214	20,606 (2801; 38,566)	0.93 (0.13; 1.74)	6.70%	16.97
	Influenza	7677	21,021 (14,826; 27,300)	0.95 (0.67; 1.23)	6.84%	2.74
70–74	RSV	1302	19,915 (NC; 50,958)	1.14 (NC; 2.92)	6.23%	15.30
	Influenza	8012	22,630 (11,972; 33,527)	1.30 (0.69; 1.92)	7.08%	2.82
75–79	RSV	1383	24,986 (8158; 41,932)	1.94 (0.63; 3.26)	6.81%	18.07
	Influenza	8968	33,044 (27,120; 39,030)	2.57 (2.11; 3.03)	9.01%	3.68
80–84	RSV	1887	51,640 (22,374; 81,165)	4.81 (2.08; 7.56)	10.49%	27.37
	Influenza	11,927	51,778 (41,450; 62,244)	4.82 (3.86; 5.80)	10.52%	4.34
85–89	RSV	2202	71,082 (39,209; 103,232)	9.42 (5.19; 13.67)	13.45%	32.28
	Influenza	12,407	62,110 (50,785; 73,582)	8.23 (6.73; 9.75)	11.75%	5.01
≥ 90	RSV	2136	67,520 (30,335; 105,115)	14.38 (6.46; 22.38)	14.16%	31.61
	Influenza	10,744	58,618 (45,345; 72,111)	12.48 (9.66; 15.35)	12.30%	5.46

*CI* confidence intervals, *LRTD* lower respiratory tract disease, *NC* not calculated, *RSV* respiratory syncytial virus

^a^Percentage attributable is the total predicted number (n) for each pathogen (RSV or influenza) divided by the total predicted ARI admissions across the study period. Values < 0 are denoted as ‘NC’

A sensitivity analysis was conducted using an expanded definition of ARI hospital admissions, including additional ICD-10 codes for related cardiac and respiratory conditions (Table 3). This analysis yielded considerably higher rates of RSV-related cardiorespiratory hospital admissions compared to influenza-related admissions, but the pattern of increasing hospitalisation rates with increasing age was maintained. For instance, estimated cardiorespiratory hospital admission rates ranged from 2.03 (95% CI: 0.15–3.94) per 1000 person-years for RSV and 0.45 (95% CI: not calculated [NC]–1.26) per 1000 person-years for influenza in the 50–54 age group to 48.45 (95% CI: 37.08–59.94) and 17.06 (95% CI: 12.07–22.14) per 1000 person-years for RSV and influenza, respectively, in the ≥ 90 years age group (Table 3).

**Table 3:** Total number and annual rate of RSV-associated and influenza-associated cardiorespiratory hospital admissions among adults aged ≥ 50 years

Age group	Pathogen	Observed hospital admissions, n	Total predicted hospital admissions, n (Exact Poisson CI)	Predicted admission rates per 1000 person-years (Exact Poisson CI)
Overall ≥ 50	RSV	11,812	1,417,611 (744,612; 2,099,843)	9.48 (4.98; 14.04)
	Influenza	133,309	494,416 (207,479; 787,632)	3.31 (1.39; 5.27)
Overall ≥ 60	RSV	10,822	1,277,210 (715,382; 1,846,424)	12.90 (7.22; 18.64)
	Influenza	118,724	448,692 (208,722; 693,689)	4.53 (2.11; 7.00)
50–54	RSV	364	52,065 (3858; 101,217)	2.03 (0.15; 3.94)
	Influenza	5657	11,455 (NC; 32,215)	0.45 (NC; 1.26)
55–59	RSV	626	85,908 (18,360; 154,660)	3.46 (0.74; 6.23)
	Influenza	8928	33,593 (4954; 63,043)	1.35 (0.20; 2.54)
60–64	RSV	956	120,170 (32,401; 209,484)	5.10 (1.37; 8.89)
	Influenza	11,299	44,873 (7715; 83,074)	1.90 (0.33; 3.52)
65–69	RSV	1164	179,991 (65,595; 296,430)	8.13 (2.96; 13.39)
	Influenza	13,455	66,730 (18,191; 116,642)	3.01 (0.82; 5.27)
70–74	RSV	1248	167,234 (63,470; 272,769)	9.57 (3.63; 15.61)
	Influenza	14,277	67,347 (23,178; 112,718)	3.85 (1.33; 6.45)
75–79	RSV	1352	156,064 (63,195; 250,267)	12.13 (4.91; 19.46)
	Influenza	16,074	54,761 (15,386; 95,045)	4.26 (1.20; 7.39)
80–84	RSV	1821	191,778 (107,348; 277,181)	17.86 (9.99; 25.81)
	Influenza	21,704	60,597 (24,662; 97,198)	5.64 (2.30; 9.05)
85–89	RSV	2146	226,179 (163,287; 289,672)	29.96 (21.63; 38.37)
	Influenza	22,636	72,992 (45,923; 100,475)	9.67 (6.08; 13.31)
≥ 90	RSV	2135	227,525 (174,161; 281,490)	48.45 (37.08; 59.94)
	Influenza	19,279	80,132 (56,679; 103,998)	17.06 (12.07; 22.14)

*ARI* acute respiratory infection, *CI* confidence intervals, *ICD-10* International Classification of Diseases, 10th Revision, *NC* not calculated, *RSV* respiratory syncytial virus

Sensitivity analysis. Cardiorespiratory admissions are an expanded definition of ARI hospital admissions by inclusion of additional ICD-10 codes for related cardiac and respiratory conditions (ICD-10 codes I00-99 and J00-99)

### Observed in-hospital mortality

When looking at ICD-10 coded in-hospital mortality from ARI, mortality rates per 1000 person-years were highest in the older age groups, as anticipated. Overall ARI observed in-hospital mortality rate increased from 1.66 per 1000 person-years in the 50–54 age group to 89.50 per 1000 person-years in those aged ≥ 90 years. For pathogen-specific ARIs, in-hospital mortality increased from 0.97 per 1000 person-years in the 50–54 age group to 26.07 per 1000 person-years in those aged ≥ 90 years.

### Time-series modelling-estimated in-hospital mortality

RSV and influenza were associated with remarkably similar estimates of LRTD in-hospital mortality (Table 4). The modelling analysis estimated annual in-hospital mortality rates of 0.19 per 1000 person-years (95% CI: 0.05–0.32) for RSV and 0.19 per 1000 person-years (95% CI: 0.14–0.23) for influenza in the overall population. This rate increased with age, reaching 1.81 (95% CI: 0.40–3.24) and 2.03 (95% CI: 1.53–2.54) per 1000 person-years for RSV and influenza, respectively, in adults aged ≥ 90.

**Table 4:** Total number and annual rate of RSV-associated and influenza-associated LRTD in-hospital deaths among adults aged ≥ 50 years

Age group	Pathogen	Observed in-hospital deaths, n	Total predicted in-hospital deaths, n (Exact Poisson CI)	Predicted rates per 1000 person-years (Exact Poisson CI)
Overall ≥ 50	RSV	919	27,847 (7840; 48,044)	0.19 (0.05; 0.32)
	Influenza	6849	27,857 (20,911; 34,902)	0.19 (0.14; 0.23)
Overall ≥ 60	RSV	868	26,009 (6551; 45,658)	0.26 (0.07; 0.46)
	Influenza	6389	26,807 (20,043; 33,672)	0.27 (0.20; 0.34)
≥ 90	RSV	228	8500 (1889; 15,201)	1.81 (0.40; 3.24)
	Influenza	1459	9538 (7182; 11,941)	2.03 (1.53; 2.54)

*CI* confidence intervals, *LRTD* lower respiratory tract disease, *RSV* respiratory syncytial virus.

A sensitivity analysis was also conducted on the time-series modelling of in-hospital deaths, to estimate the total number and annual rate of RSV- and influenza-associated cardiorespiratory in-hospital mortality (Supplementary Table 1). Similar to the pattern observed for hospital admissions, rates of in-hospital cardiorespiratory mortality for RSV were consistently higher compared with influenza. For the overall population aged ≥ 50 years, the rate of RSV-related cardiorespiratory in-hospital mortality was 0.94 per 1000 person-years (95% CI: 0.58–1.30), while influenza-related in-hospital mortality was 0.23 (95% CI: 0.12–0.35) per 1000 person-years. In the ≥ 90 population, these rates increased to 8.47 (95% CI: 4.31–12.68) and 2.70 (95% CI: 1.35–4.08) per 1000 person-years for RSV and influenza, respectively.

### Healthcare resource utilisation

#### Length of hospital stay

**Fig. 2:**
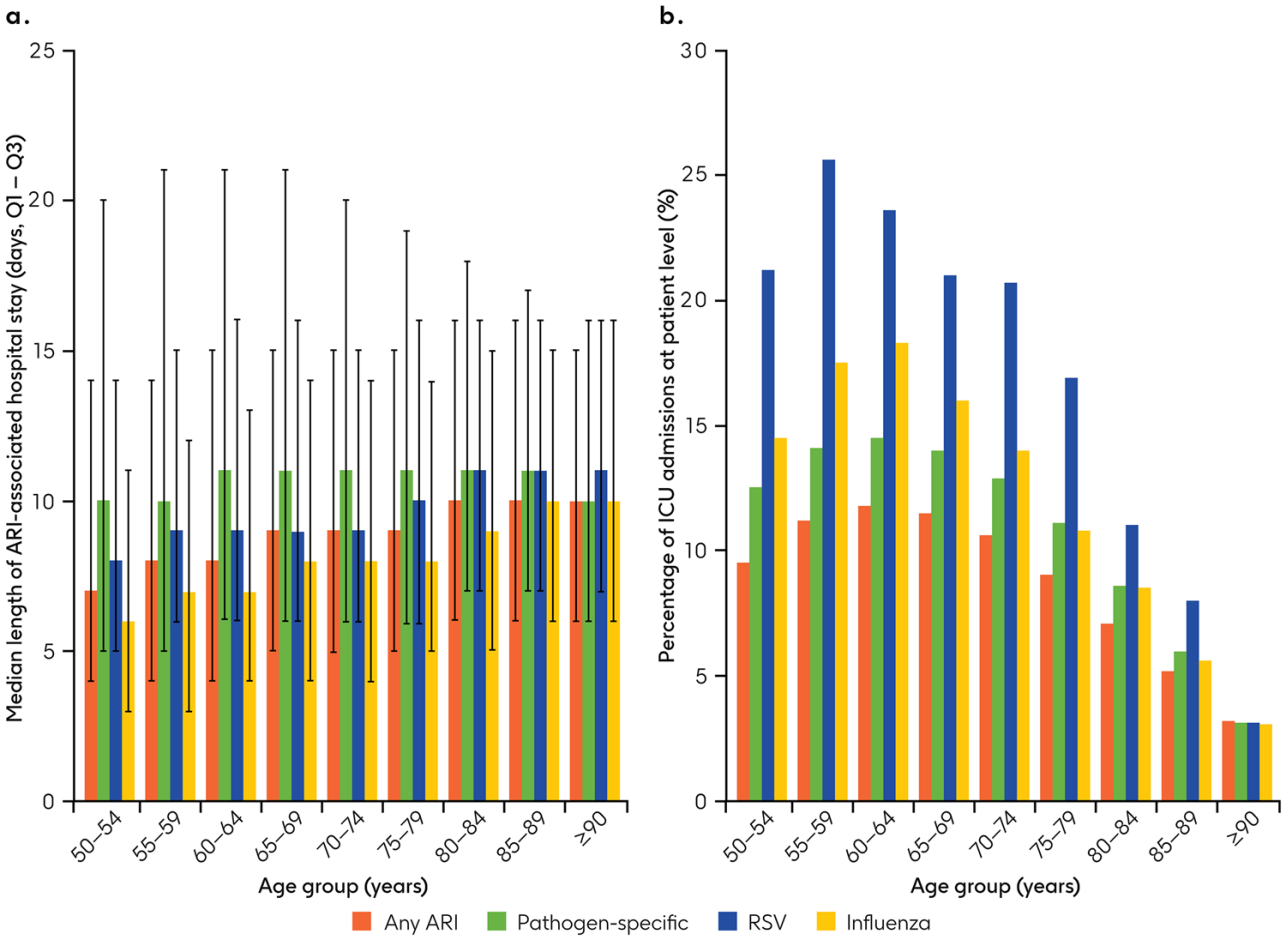
Median length of ARI-associated hospital stay (**a**) and percentage of patients admitted to ICU (**b**), by age and diagnosis group. *ARI* acute respiratory infection, *ICU* intensive care unit, *Q1* first quartile, *Q3* third quartile, *RSV* respiratory syncytial virus

The median length of stay for ARI-associated inpatient admissions remained relatively stable across age groups, with slight variations observed by diagnosis group (Fig. 2a).

For any ARI, the median (interquartile range [IQR]) length of stay ranged from 7.0 (4.0, 14.0) days in the 50–54 age group to 10.0 (6.0, 15.0) days in individuals aged ≥ 90 years. Pathogen-specific ARI showed a slightly higher median in the youngest age group (10.0 [5.0, 20.0] days for 50–54-year-olds) and was stable across ages.

RSV-associated admissions had a median length of stay of 8.0–11.0 days across age groups, with IQRs ranging between 5.0–16.0 days. Influenza-related hospitalisations were comparable, with a median length of stay of 6.0–10.0 days across age groups (Fig. 2a).

#### Hospital readmission

High levels of all-cause readmission following RSV-coded ARI admissions were found, with 39.6% of all adults aged ≥ 50 being readmitted within 90 days of an RSV-coded hospitalisation. The proportion of patients undergoing all-cause readmission decreased in older age groups, with 32.3% of RSV-associated patients ≥ 90 years old being readmitted within 90 days of an RSV diagnosis. The proportion of patients readmitted for ARI peaked in the 60–64 years age group for RSV, reaching 21.5% of patients.

For influenza-coded ARI, a similar pattern was observed, with 34.6% of all patients aged ≥ 50 being readmitted within 90 days of an influenza admission, compared with 30.3% of those aged ≥ 90 years. For influenza, the highest proportion of ARI-associated readmission occurred in those aged 85–89 years, reaching 14.2%.

#### ICU admissions

The percentage of ARI patients with an ICU admission steadily declined with increasing age. For example, 9.5% of ARI patients with any ARI in the 50–54 years age group had an ICU admission, in comparison to only 3.2% in the ≥ 90 years age group. Notably, ICU admissions for RSV-specific ARI were highest in the 55–59 years age group (25.6%) and declined progressively, reaching 3.1% among individuals aged ≥ 90 years (Fig. 2b).

## Discussion

By leveraging real-world data from the PMSI database and applying time-series modelling, we estimated the annual rates of RSV- and influenza-related hospital admissions and in-hospital deaths among adults aged ≥ 50 years in France. Additionally, RSV- and influenza-associated healthcare resource utilisation was also investigated in this population. The data presented herein demonstrate that patients hospitalised with RSV in France tend to be older and have pre-existing chronic conditions including cardiac and respiratory diseases. The data further show a substantial burden of RSV-related hospital admission, remarkably similar to that of influenza, which increases considerably with increasing age. Furthermore, RSV was found to be associated with very high rates of cardiorespiratory-related hospitalisation and mortality. Importantly, the application of TSM revealed extensive underreporting of RSV-related hospital admissions in France.

RSV was found to be a greater burden in older adults and those with chronic conditions. A key observation from this study was the high prevalence of immunocompromised individuals among ARI admissions, particularly for RSV-specific infections. This aligns with prior research showing that patients with haematologic malignancies, solid organ transplants or those on immunosuppressive therapy are more vulnerable to severe respiratory infections [1, 26].

This study also found a large relative increase with age in RSV-associated (observed and predicted) hospital admissions and mortality. The large difference between observed and predicted admissions for RSV highlights the substantial underreporting and underdiagnosis of RSV in France. Recent work across Europe has highlighted that adjusting for case underreporting results in RSV-associated hospitalisation rates that are 2.2 to 6.4 times higher than those typically reported [15]. Adjusted hospitalisation rates among older adults across five European countries ranged from 193 to 414 per 100,000 person-years [15], consistent with the rates reported herein. In France, a study comparing the results of RSV virological testing with the number of RSV-coded hospitalisations among adults aged ≥ 65 at two hospitals between 2017 and 2022 calculated a ‘correction factor’ of between 4.00 and 5.22 across years, consistent with the European range [8]. This resulted in overall estimated RSV-associated hospitalisation rates of 55.17 to 136.75 per 100,000 adults aged ≥ 65 years nationwide [8]. The present study provides more recent and detailed estimates of the RSV-associated disease burden among older adults in France.

The results of this study must be interpreted in the context of previous literature. A previous study used modelling approaches and data from six countries in Europe to extrapolate the rate of RSV-associated hospital admissions to 28 European countries, including France [27]. That analysis yielded rates per 1000 adults (95% CI) of 0.70 (0.53, 0.88), 1.90 (1.60, 2.19) and 3.01 (2.18, 3.85) for adults aged 65–74, 75–84 and ≥ 85 years old in France, respectively [27]. When compared to the present results, the use of TSM yielded higher rates among those aged 80–89 and ≥ 90 years old, but the overall trend of increasing hospitalisation with increasing age is similar. Another recent study compared the burden of RSV infection in France with that of influenza. In adults aged ≥ 65 years, the mean length of hospitalisation was 12.1 days for RSV and 10.7 days for influenza [2]. In the present study, RSV-related hospital stays were also consistently longer than influenza-related stays across all age groups. Nuttens and colleagues also reported higher rates of ICU admission among RSV-hospitalised patients compared with patients with influenza [2], consistent with the present study. Loubet and colleagues analysed data from the PMSI database between 2012 and 2021 to assess the burden of RSV infections in hospitalised adults [6]. Among patients with a mean age of 74.1 years, they found mean length of RSV hospital stay to be 12.3 days, with 10.9% of patients being admitted to intensive care. In-hospital mortality was calculated as 7.3% overall and 8.1% in adults ≥ 60 years old [6]. This is consistent with the increase in predicted rate of in-hospital deaths with increasing age observed in the present study.

Another key finding of this study is the similar mortality rates between RSV and influenza. By applying TSM, the annual mortality rates for each disease reached 0.19 per 1000 person-years. Not only this, but RSV was also found to consistently lead to higher rates of cardiorespiratory-related events, compared with influenza. In adults aged ≥ 50 years old, TSM predicted almost 1.5 million RSV-attributable cardiorespiratory hospital admissions, compared with approximately 500,000 for influenza, equating to rates of 9.5 and 3.3 per 1000 person-years for the two viruses, respectively. Prior research has indicated a strong link between RSV and cardiovascular events [28]. In a previous study of adults aged ≥ 60 years old in Spain, estimated RSV-attributed cardiorespiratory hospitalisations ranged from 4.38 to 4.76 per 1000 person-years, increasing to 13.25 to 15.06 per 1000 person-years for those older than 80 years [29]. In the UK, a considerable number of cardiorespiratory hospitalisations has also been reported among patients with RSV infection, ranging from 18 per 100,000 people to 174 per 100,000 people in the 50–64 and ≥ 75 year old age groups, respectively [13]. These findings highlight the research value of using broader cardiorespiratory definitions to account for potential misclassification of respiratory pathogens in hospital discharge data.

Readmissions following the first RSV-attributed hospitalisation were high, with results aligning to research suggesting that patients may experience an increase in frailty following RSV infection, leading to increased dependency on healthcare systems, exemplified by Branche and colleagues [30]. This demonstrates that indirect or direct complications following RSV infection must be considered in the development of RSV preventative strategies. There exists a disproportionate impact on older populations, with the associated economic burden continuing years after infection.

The proportion of patients requiring ICU admission following RSV infection was also high, exceeding that of patients infected with influenza and previously-reported data on ICU admission among adult patients with RSV in France [6]. Compared with the population of patients with influenza-specific ARI in this study (n = 97,109), relatively few patients with RSV-specific ARI were identified (n = 7459). Given that RSV-coded hospital admissions are a considerable underestimate of the total number of RSV-attributable cases, this discrepancy may reflect the current landscape of RSV diagnosis, whereby patients presenting with severe infection may be the most likely to be diagnosed with RSV. If this is indeed the case, it would be expected that a higher proportion of hospitalised patients would require ICU admission, as patients with less severe RSV infection may be un- or misdiagnosed. Additionally, other studies have reported ICU admission in up to roughly one quarter of adult RSV cases, consistent with the results from this study [21, 31, 32]. In France, chronic heart failure, chronic respiratory failure and chronic obstructive pulmonary disease were identified as significant risk factors for ICU admissions among patients with RSV [32]. Given that the proportion of patients with chronic respiratory/pulmonary conditions and cardiac disorders was highest among those with RSV-specific ARI in the present study, this may also explain the increased proportion of ICU admissions among patients with RSV compared to influenza.

This study is associated with some limitations. The reliance on hospital admission data may underestimate the true burden of RSV and influenza, as older adults and high-risk individuals may receive treatment in outpatient or primary care settings, rather than in hospitals. Moreover, the study is dependent on ICD-10 coding, which may lead to misclassification of cases, particularly when distinguishing between viral and bacterial ARI. Furthermore, while external data sources were used for model calibration, the accuracy of predictions may still be subject to data completeness and reporting biases. In particular, the use of paediatric data as a proxy for RSV infection in older adults may have led to under- or overestimation of RSV-attributable hospitalisations in adults, as the correlation between the two populations may not be linear or constant. Future research should explore integrated surveillance approaches that combine hospital, outpatient and virological data to refine estimates of disease burden.

Nonetheless, this study provides a comprehensive evaluation of RSV- and influenza-associated hospital admissions among adults aged ≥ 50 years in France. The findings presented herein confirm that RSV is a major contributor to acute respiratory morbidity and mortality in older adults, while revealing the extent to which reliance on diagnostic coding results in underestimation of RSV-associated hospitalisation. Overall, this study highlights the substantial burden of RSV in older populations within France, demonstrating an urgent need to prioritise RSV prevention alongside influenza and other causes of LRTD.

## Supplementary Information

Below is the link to the electronic supplementary material.

Supplementary file1 (XLSX 71 KB)

Supplementary file2 (DOCX 74 KB)

## Data Availability

Data were extracted from the nationwide hospitalisation database (PMSI), which comprehensively and exhaustively records all hospitalisations in France across all types of care facilities for reimbursement purposes. Data used for this publication were generated by IQVIA. For access to anonymised subject level data, please contact IQVIA.
