# Supplementary material for: Respiratory syncytial virus in adults aged ≥ 50 years in France: a database and time-series modelling analysis (2014–2020)
Source: Infection. 2026 Jun 1;54(4):1825–37. doi: 10.1007/s15010-026-02799-7 (PMC13476349; doi:10.1007/s15010-026-02799-7)
Supplement: Supplementary file 1 [file 15010_2026_2799_MOESM2_ESM.docx]

**SUPPLEMENTARY MATERIALS**

**Supplementary Table 1** Total number and annual rate of RSV-associated and influenza-associated cardiorespiratory in-hospital deaths among adults aged ≥50 years

| **Age group** | **Pathogen** | **Observed  in-hospital deaths, n** | **Total predicted in-hospital deaths, n (Exact Poisson CI)** | **Predicted rates per 1,000 person-years (Exact Poisson CI)** |
| --- | --- | --- | --- | --- |
| Overall ≥50 | RSV | 875 | 139,857 (86,483; 193,648) | 0.94 (0.58; 1.30) |
|  | Influenza | 9,517 | 35,030 (18,265; 52,018) | 0.23 (0.12; 0.35) |
| Overall ≥60 | RSV | 830 | 133,951 (81,750; 186,577) | 1.35 (0.83; 1.88) |
|  | Influenza | 8,952 | 34,249 (17,827; 50,898) | 0.35 (0.18; 0.51) |
| ≥90 | RSV | 225 | 39,760 (20,222; 59,556) | 8.47 (4.31; 12.68) |
|  | Influenza | 2,242 | 12,701 (6,360; 19,178) | 2.70 (1.35; 4.08) |

Sensitivity analysis. **CI**, confidence intervals; **RSV**, respiratory syncytial virus.

**Supplementary Fig. 1** Final model observed vs predicted hospitalisations for LRTD-associated hospital admissions among adults aged ≥50 years

The final model was selected based on the lowest qAIC value and included external surveillance data as predictors for RSV and influenza. This model incorporated a 4-week lag for Géodes-observed RSV data, no lag for Géodes-observed influenza data and seasonal trends (model fit AIC=14,213). The model based on all patients aged ≥50 years was applied for all other age groups. **AIC**, Akaike Information Criterion; **ARI**, acute respiratory infection; **qAIC**, quasi-Akaike Information Criterion; **RSV**, respiratory syncytial virus.

**Supplementary File 1** List of ICD-10 codes used to define diagnostic groups and comorbidities
